# The Microbiome of the Gastrointestinal Tract of a Range-Shifting Marine Herbivorous Fish

**DOI:** 10.3389/fmicb.2018.02000

**Published:** 2018-08-28

**Authors:** Jacquelyn Jones, Joseph D. DiBattista, Michael Stat, Michael Bunce, Mary C. Boyce, David V. Fairclough, Michael J. Travers, Megan J. Huggett

**Affiliations:** ^1^Centre for Marine Ecosystems Research, School of Science, Edith Cowan University, Joondalup, WA, Australia; ^2^Trace and Environmental DNA Laboratory, School of Molecular and Life Sciences, Curtin University, Perth WA, Australia; ^3^Australian Museum Research Institute, Australian Museum, Sydney, NSW, Australia; ^4^Department of Biological Sciences, Macquarie University, Sydney, NSW, Australia; ^5^Centre for Ecosystem Management, School of Science, Edith Cowan University, Joondalup, WA, Australia; ^6^Department of Primary Industries and Regional Development, Fisheries Division, Government of Western Australia, Hillarys, WA, Australia; ^7^School of Environmental and Life Sciences, University of Newcastle, Ourimbah, NSW, Australia

**Keywords:** fish gut microbiota, Siganidae, marine heat waves, short chain fatty acids (SCFA), marine herbivory

## Abstract

Globally, marine species’ distributions are being modified due to rising ocean temperatures. Increasing evidence suggests a circum-global pattern of poleward extensions in the distributions of many tropical herbivorous species, including the ecologically important rabbitfish *Siganus fuscescens*. Adaptability of a species to such new environments may be heavily influenced by the composition of their gastrointestinal microbe fauna, which is fundamentally important to animal health. *Siganus fuscescens* thus provides an opportunity to assess the stability of gastrointestinal microbes under varying environmental conditions. The gastrointestinal microbial communities of *S. fuscescens* were characterized over 2,000 km of Australia’s western coast, from tropical to temperate waters, including near its current southern distributional limit. Sequencing of the 16S rRNA gene demonstrated that each population had a distinct hindgut microbial community, and yet, 20 OTUs occurred consistently in all samples. These OTUs were considered the ‘core microbiome’ and were highly abundant, composing between 31 and 54% of each population. Furthermore, levels of short chain fatty acids, an indicator of microbial fermentation activity, were similar among tropical and temperate locations. These data suggest that flexibility in the hindgut microbiome may play a role in enabling such herbivores to colonize new environments beyond their existing range.

## Introduction

One of the most realized predictions of global climate change is poleward range-shifts in species distributions as their range margins are largely dictated by their physiological tolerance to physical and abiotic environmental conditions (Sorte et al., 2010). For example, temperature limits the range of most animal species due to associated well-defined physiological thresholds (for review see Bates et al., 2013). This is especially true for aquatic ectotherms, as their metabolism, growth and maturation rates are influenced by temperature (Lek et al., 2012; Trip et al., 2014). Over the last 50 years, water temperatures off the west coast of Australia have been increasing faster than the global average, resulting in noticeable ecological modifications, including the loss of diversity of temperate invertebrates, algae and demersal fishes from the northern limits of their distribution (Cheung et al., 2012; Wernberg et al., 2013, 2016). During austral summer in 2010/11, the west coast of Australia also experienced a ‘marine heat wave,’ with peak ocean temperatures reaching ∼5°C above the average and extending from the coast up to 200 km offshore (Pearce et al., 2011). The sustained high temperature resulted in a number of significant impacts to marine biota including mortalities of fish, prawns (shrimp), abalone and the appearance of certain tropical species in more temperate environments (Pearce and Feng, 2013; Caputi et al., 2016).

The fitness of a species to changing environmental conditions is partially reliant upon host-associated microbial communities (El Kafsi et al., 2016; Peixoto et al., 2017). For vertebrates, the microbiota – and their collective genes known as the microbiome – provide the host with an extended genome, which greatly increases their potential functionality (Hacquard et al., 2015). In particular, microbes closely associated with the organs throughout the gastrointestinal (GI) system not only contribute to nutrient acquisition, but increase host resilience to GI pathogens through stimulation of the host immune system and secretion of antimicrobial compounds (Hacquard et al., 2015). Microbes of the GI system have been most studied in mammals, however, insects and fish are now of great interest (Sanders et al., 2014; Ghanbari et al., 2015), given that these represent the most speciose invertebrate and vertebrate taxa on the planet, respectively. Research on marine fish herbivory has focused primarily on feeding habits and grazing rates rather than post-ingestive processes (Clements and Choat, 1997; Miyake et al., 2015); and of the limited research that has been conducted on post-ingestive processes, most have focused on gut morphology rather than the functional role of GI microbes (Clements et al., 2009). However, molecular based studies have reported a large variety of microorganisms within the GI tracts of fish, and that their community diversity increases from carnivorous fish to herbivorous fish (Wang et al., 2017).

Marine herbivorous fish consume seagrasses and/or algae, which differ biochemically in their structure and in the production of secondary metabolites (Puk et al., 2015). Microbes enhance the digestive capabilities and contribute greatly to the biochemical tolerance of their host through the production of exogenous enzymes. For example, some bacteria isolated from the GI tract of marine fish such as Atlantic cod (*Gadus morhua*), Atlantic salmon (*Salmo salar*), sea bass (*Dicentrarchus labrax*), gray mullet (*Mugil cephalus*) and pinfish (*Lagodon rhomboides*) produce either amylase, proteases or cellulase (Ray et al., 2012). Nutrients made available to the host via microbial fermentation are predominantly in the form of short chain fatty acids (SCFA), of which, acetic, propionic and butyric acids are the most common. SCFA that are absorbed by the host are thought to activate host cell signaling pathways, mediate the production of gut hormones that reduce food intake and can affect the physiology of the hindgut. Cross-feeding of SCFA between different members of the microbial community is also an important driver of community composition (Ríos-Covián et al., 2016). As many fish species are of significant economic and ecological importance, most of the information about GI microbial mediation of nutritional ecology in fish has come from aquaculture studies (Tarnecki et al., 2017). To fully understand the aquatic systems on which we depend, the role of the microbiome in wild populations of fish must also be investigated. Indeed, there is an urgent need to understand both baseline associations and the possible impacts of marine disturbance events such as rapid temperature driven range-shifts.

Applying next-generation sequencing to understand GI microbial communities in fish is a recently developed area of research. As such, there are inconsistencies with the approach that is used to sample these communities, with differences such as sampling procedure, sample storage, DNA extraction protocol and sample type varying between studies. In particular, for GI microbial communities, the gut region or length of gut material collected, as well as the component of the gut (gut wall or gut contents) will impact the data obtained from the samples. For example, some studies do not consider specialization in microbial community composition along the GI tract and instead homogenize the midgut and hindgut together (Xia et al., 2014; Givens et al., 2015; Miyake et al., 2015; Liu et al., 2016). Those studies that have examined material separately from two or more regions of the gut provide evidence for specialization along the GI tract (Ye et al., 2014; Nielsen et al., 2017). The upper gut regions appear to host a higher abundance of microbes associated with sediment, food sources and water (most notably Cyanobacteria) as opposed to hindgut microbes that are associated with the fermentation process, such as *Clostridium* and Bacteroidetes (McDonald et al., 2012; Sanchez et al., 2012; Ye et al., 2014). Finally, the midgut contents have been shown to contain a more transient microbial community than both the hindgut contents and the wall of the intestine (Nielsen et al., 2017). Given these apparent differences, it is important to evaluate microbial community structure along the gut in order to determine the most appropriate section of the gut to use for GI microbial community studies.

The tropical rabbitfish *Siganus fuscescens* (Houttuyn, 1782) is one species that may be expanding its poleward range limit further into temperate waters on both the east and west coasts of Australia (Vergés et al., 2014; Lenanton et al., 2017; Zarco-Perello et al., 2017). There is evidence that this species may be self-recruiting and occurring in greater numbers in recent years at the poleward margin of its historical distribution (∼32°S) on the west coast of Australia (Lenanton et al., 2017; Zarco-Perello et al., 2017). Also, higher feeding rates have been associated with the recent increase in abundance of tropical herbivores including *S. fuscescens* on temperate reefs of Western Australia, causing declines in the biomass of habitat-forming kelp (Bennett et al., 2015; Zarco-Perello et al., 2017) and seagrasses (Hyndes et al., 2016). Despite the importance of this species to the wider ecological setting of the temperate reefs of Western Australia, the impact of exposure to varying environmental factors on its GI microbial community is unknown. Here, we tested the general hypothesis that microbial community structure and diversity shifts from a characteristic composition of microbes in fish from long-established populations, to a more variable composition within fish from populations in the newly established range. Specifically, we tested whether: (1) the microbial communities in the midgut and hindgut of *S. fuscescens* differ, and hence should be sampled separately; (2) microbial community alpha- and beta-diversity within populations of *S. fuscescens* residing in four populations from 31 to 16°S latitude differ; (3) quantities of SCFA located in the hindgut of the one *S. fuscescens* population from the traditional range differed from one population from the newly established range and, (4) if there was evidence of a core gut microbiome in wild populations of *S. fuscescens* across a large geographic distance.

## Materials and Methods

### Sample Collection and DNA Extraction

**FIGURE 1:**
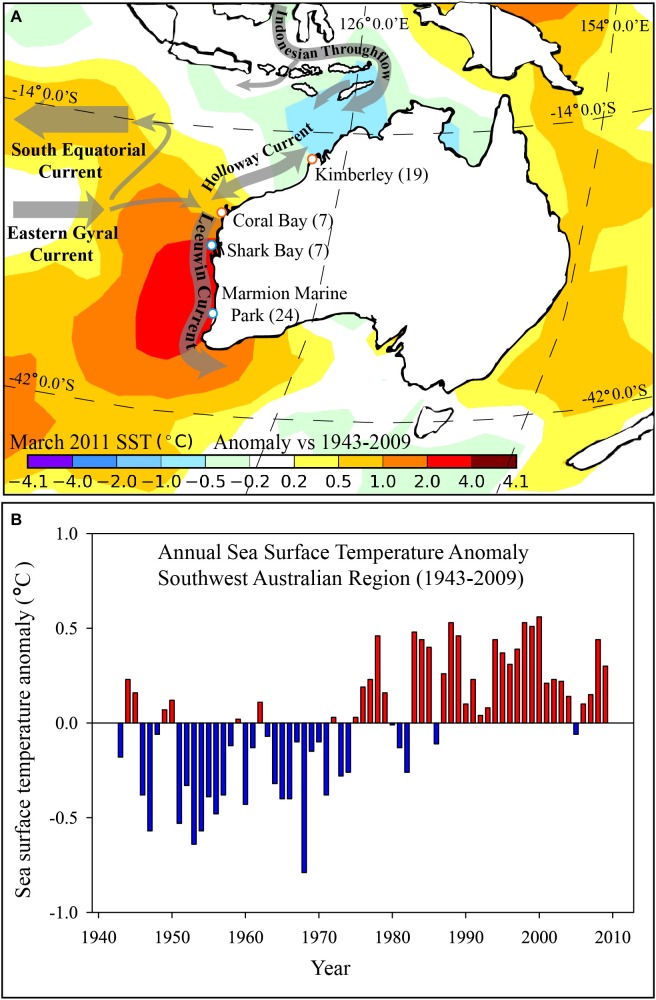
**(A)** Map showing sampling sites (white dots) for *Siganus fuscescens* along the west coast of Australia and the spatially smoothed mean sea surface temperature (SST) anomaly (°C) for March 2011 versus the 1943–2009 period for the region; and **(B)** Annual SST anomaly for the south-west region of Australia between 1943 and 2009 (based on departures from the 1961–1990 average). **(A)** Generated from the 1° “Reynolds” dataset (Reynolds et al., 2002) and annual time series data for panel. Numbers of fish collected at each site are shown in brackets. **(B)** Calculated from the NOAA Extended Reconstructed Sea Surface Temperature Version 5 (ERSST v5, Huang et al., 2017) and plotted for the south-west marine region of Australia by the Australian Bureau of Meteorology.

*Siganus fuscescens* were collected from the coast of Western Australia in the austral summer of 2016 (January) from the temperate waters of Marmion Marine Park (31°48′15.49″S, 115°43′6.67″E, 23.1°C, 5 m depth, *N = 22*), and subtropical waters of Coral Bay at the southern end of Ningaloo Reef (23°10′58.14″S, 113°45′38.62″E, 24.7°C, 5 m depth, *N = 7*), in October 2016 from the subtropical waters of Shark Bay (26°01′47.28″S, 113°33′12.49″E, 26.6°C, 2 m depth, *N = 6*), and from the tropical waters around the Lacepede Islands in the Kimberley Bioregion (*as per* IMCRA; Thackway and Cresswell, 1998) in August 2016 (16°51′14.57″S, 122°10′39.45″E, 26.9°C, 5m depth, *N =* 16) (**Figure 1**). Samples were collected in Marmion Marine Park and Coral Bay during field work dedicated to the collection of these samples only. Fish from the other two sites were obtained opportunistically through work being done in those sites by the Fisheries Division of the Department of Primary Industries and Regional Development, Western Australia, for other projects. Therefore, the samples were collected across a 10 month period and at Shark Bay and the Kimberley, only fish samples (and not water samples) were collected due to time constraints. Whole specimens were collected by hand spear or trap, immediately placed on ice and either frozen or processed within 12 h of collection. All procedures were approved by the Animal Ethics Committee at Curtin University (AEC_2015_27) to J. D. DiBattista. Only fish measuring > 24 cm in total length (assumed to be adults, **Supplementary Table 1**) were used. Fish with damaged guts were excluded and the GI system was dissected out from individual fish. There was no specialization or differentiation in morphology along the length of the gut. Therefore, we used a conservative approach and here refer to the midgut as the region directly behind the stomach, and the hindgut as the region at the terminal end of the gut. Gut material (∼1 g) was squeezed out and collected into separate, aseptic 75% ethanol suspensions from the midgut (approximately 1 – 4 cm behind the stomach), (Marmion Marine Park and Coral Bay only) and the hindgut (approximately 1 – 4 cm from the terminal end, all sites). Midgut and hindgut samples were immediately frozen at -80°C. This method was selected as it has previously been successful in determining differences in gut microbiomes of wild caught fish for comparisons between sections of the gut within a single fish and between different species of fish (e.g., Clements et al., 2007; Miyake et al., 2015). This method is likely to have captured both transient and symbiotic members of the GI microbiome community, while sampling of the gut wall targets less transient members (Nielsen et al., 2017). The gut wall was not sampled in our study.

To provide a background profile of the environmental microbial communities at different latitudes, three 1 L water samples were collected at the same time as fish samples were collected from sites in Coral Bay and Marmion Marine Park. Seawater samples were placed on ice and filtered via peristalsis over a 0.2 μm filter membrane, within 6 h of collection. Filter membranes were frozen immediately in liquid nitrogen and stored at -80°C. Filter membranes (from water samples) and approximately 0.5 g of homogenized gut contents from each specimen (**Supplementary Table 2**) were used to extract total genomic DNA using the PowerSoil Kit (Mo Bio Laboratories, Carlsbad, CA, United States) according to the manufacturers’ protocol.

### Characterization of the Microbial Community via 16S rRNA MiSeq Illumina Sequencing

Bacterial sequencing from water (*N* = 6), as well as midgut (*N* = 31) and hindgut (*N* = 57) samples from *S. fuscescens* was performed on an Illumina MiSeq platform (Illumina, San Diego, CA, United States) located in the Trace and Environmental DNA (TrEnD) laboratory at Curtin University. The V4 region of the 16S rRNA gene was targeted using the primer pair 515F (5′ GTGBCAGCMGCCGCGGTAA 3′) and 806R (5′ GGACTACHVGGGTAWTCTAAT 3′) (Caporaso et al., 2012). Initially, the optimal yield of DNA to be added to each PCR reaction was determined using qPCR (Murray et al., 2015). Amplicons for Illumina sequencing were generated using a single round of PCR and fusion tag primers consisting of Illumina adaptor regions, MID tags unique to each sample and the 16S rRNA template specific primers. PCR reagents included 1 × AmpliTaq Gold^®^ Buffer (Life Technologies, Carlsbad, CA, United States), 2 mM MgCl_2_, 0.25 μM dNTPs, 10 μg BSA, 5 pmol of each primer, 0.12 × SYBR^®^ Green (Life Technologies), 1 Unit AmpliTaq Gold DNA polymerase (Life Technologies), 2 μl of DNA and Ultrapure^TM^Distilled Water (Life Technologies) to 25 μl total volume. PCRs were run on Applied Biosystems StepOnePlus Real-Time PCR system as follows: initial denaturation at 95°C for 5 min, then 35 cycles of 30 s at 95°C, 30 s at 50°C, and 45 s at 72°C, with a final extension for 10 min at 72°C. Duplicates of each sample were combined and the samples were pooled in equimolar ratios determined by qPCR and quantification on a Labchip^®^ GX Touch HT (Perkin Elmer, Waltham, MA, United States). Extraction controls containing no tissue, and PCR negative controls, were added to sequencing runs to assess cross-contamination. Final sequencing libraries were size selected using a Pippin Prep (Sage Science, Beverly, MA, United States), purified using the Qiaquick PCR purification Kit (Qiagen, Venlo, Netherlands) and sequenced uni-directionally using Illumina 300 cycle MiSeq^®^ v2 Reagent Kits and standard flow cells.

Geneious version 8.0.5^1^ was used to extract raw data, using 100% matches to adaptor, MID tags and primer sequences. Additional quality filtering of the data was performed using MOTHUR version 1.35.0 (Schloss et al., 2009) following the MOTHUR MiSeq standard protocol (Kozich et al., 2013). Briefly, sequences less than 200 bp, or sequences with any ambiguous base calls were filtered out, and duplicates were merged. The sequences were then aligned to the SILVA reference alignment, chimeras and poorly aligned sequences were removed, and operational taxonomic units (OTUs) were defined by clustering at a 0.03 divergence (97% similarity) cut-off. Final OTUs were taxonomically classified using BLASTn against a curated database derived from SILVA, greengenes, RDP II and NCBI^2^ (DeSantis et al., 2006; Wang et al., 2007). Taxonomic identities of abundant OTUs (>1% mean relative abundance per sample type) that had been assigned to an unknown phyla were explored further by BLASTn searches against all available sequences in NCBI and by manually examining the phylogenetic comparison of sequences in arb (Ludwig et al., 2004). For this comparison the arb SilvaNR 123 database release was used (Quast et al., 2013). Alpha diversity indices (Chao index and Shannon’s H) were calculated using MOTHUR. Raw sequences have been deposited in the NCBI sequence read archive (SRA) under biosample accession numbers SAMN07425686 to SAMN07425756.

### Extraction and Quantification of Short Chain Fatty Acids

During processing, an additional 1.0 g of hindgut material was collected from each fish from Marmion Marine Park and Coral Bay. The material was placed into 1.0 mL of 0.5% analytical grade phosphoric acid (Merck; Darmstadt, Germany). Samples were frozen at -80°C, thawed, and internal standard (IS) added (50 μL of 120 mM analytical grade crotonic acid, Chemical Industries, Tokyo, Japan). Procedural blanks were also subjected to all steps alongside the extractions. Each sample and blank was homogenized and extracted twice with fresh aliquots of ethyl acetate (500 μL, Thermo Fisher Scientific; Waltham, MA, United States). The pooled ethyl acetate extracts were transferred to 2 mL vials. Calibration standards in the range 0.1–5.0 mM for propanoate, butyrate, isobutyrate, valerate and isovalerate (Merck; Darmstadt, Germany) were also prepared and IS added.

Short chain fatty acids were quantified using a TRACE 1310 gas chromatography instrument fitted with a flame ionization detector (Thermo Fisher Scientific). Separation of the analytes was achieved using a TRACE TR-wax column (60 m × 0.32 mm i.d., 1.0 μm film thickness) and helium as the carrier gas (1.7 mL min ^-1^). The column temperature gradient was 50°C (held for 2 min), increased to 120°C at a rate of 50°C/min, increased to 240°C at a rate of 10°C/min and then held at 240°C for 3 min. Injection volume was 1 μL in splitless mode. The concentration of the SCFA (millimoles g^-1^) was determined by the method of internal standards.

### Statistical Analysis

Alpha diversity of microbial communities, as assessed by the Shannon index, species richness (mean number of OTUs) and the Chao1 index, were each compared between sites using the Kruskal–Wallis test in SPSS version 23. To explore the variance in microbial assemblages, beta diversity was tested across (1) sample types (water, midgut and hindgut) and (2) sites (hindguts only) using PERMDISP and PERMANOVA in the PRIMER version 7 software, with PERMANOVA add on version 1 (Clarke et al., 2014). All data were square root transformed, to account for PCR bias, a Bray-Curtis disimilarity coefficient was used to construct a resemblance matrix and these were visualized using non-metric multi-dimensional scaling analysis. Similarity percentages (SIMPER) (Clarke, 1993) were used to identify significant differences among compositions of OTUs and which OTUs were driving patterns of separation between the factors of interest, respectively. Linear regression analysis was used to assess trends in Bray-Curtis similarity in hindgut microbial communities with distance among sites. Quantities of each amino acid (propanoate, iso-butyrate, butyrate, iso-valerate and valerate) in different sites were tested independently using *t*-tests, and as a composite using PERMANOVA.

## Results

### Comparison of Environmental, Midgut and Hindgut Microbial Communities

**FIGURE 2:**
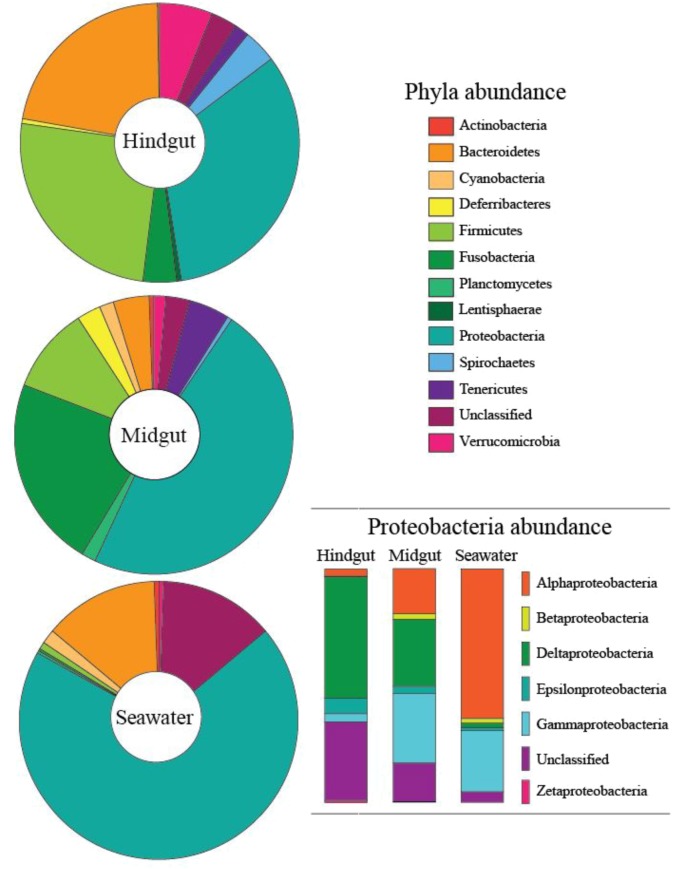
Proportion of phyla contributing more than 0.1% abundance to the microbial communities of the midgut and hindgut of *Siganus fuscescens* and nearby seawater samples from Western Australia. Insert shows the breakdown of Proteobacteria by class.

The data presented here are based on total sequences obtained following quality filtering. Subsampled (*n* = 10,761 sequences per sample) data were also analyzed and comparable results were found (data not shown). A total of 51 hindgut, 14 midgut and 6 seawater samples were successfully sequenced at the 16S rRNA gene (**Supplementary Table 2**), and binning at < 0.03% divergence resulted in 7,192 OTU assignments from 19 phyla (**Figure 2**, **Supplementary Data**, and **Supplementary Figures 1–3**). The hindgut communities, across all sampled individuals, had a mean of 937 (±7.3 SE) OTUs from 18 phyla, and were dominated by Proteobacteria (33.6%), Firmicutes (24.8%), Bacteroidetes (19.6%) and Verrucomicrobia (7.2%, **Figure 2**). Within the midgut, 18 phyla were present with an average of 480 (±17.1 SE) OTUs per sample, dominated by Proteobacteria (44.8%), Fusobacteria (25.5%), Firmicutes (10.4%) and Tenericutes (5.4%, **Table 1**). Eighteen phyla were also identified within the water communities based on an average of 1,561 (±202 SE) OTUs, and were dominated by Proteobacteria (69.8%), Bacteroidetes (13.2%) and Cyanobacteria (1.7%) (**Figure 2** and **Table 1**). Overall, 2,334 OTUs were found within the GI community that were not found within the local seawater community, and midguts had lower alpha diversity as measured by the mean number of OTUs, Shannon’s H index and the Chao1 index than hindguts (*p* < 0.05) (**Table 1**).

**Table 1:** Richness and alpha diversity estimates of the hindgut microbial communities from *Siganus fuscescens* sampled at each site in Western Australia.

Site	OTUs (*SE*)	Shannon’s *H* (*SE*)	Chao1 (*SE*)
Marmion	848.2 (17.2)	3.454 (0.03)a	3128.1 (65.4)
Shark Bay	933.3 (50.2)	3.806 (0.06)ab	3493.2 (425.5)
Coral Bay	892.7 (41.5)	3.611 (0.11)ab	2489.5 (142.7)
Kimberley	1080.5 (25.6)	3.993 (0.03)b	4333.8 (149.8)


*There were no significant differences detected for numbers of OTUs or alpha diversity between the sites, except for Shannon’s *H* index, which was lower in Marmion Marine Park than in the Kimberley Bioregion (one-factor PERMANOVA, pairwise test between Marmion and Kimberley: *t* = 2.4735, *p* = 0.018). Numbers are mean values, with standard error in brackets.*

**FIGURE 3:**
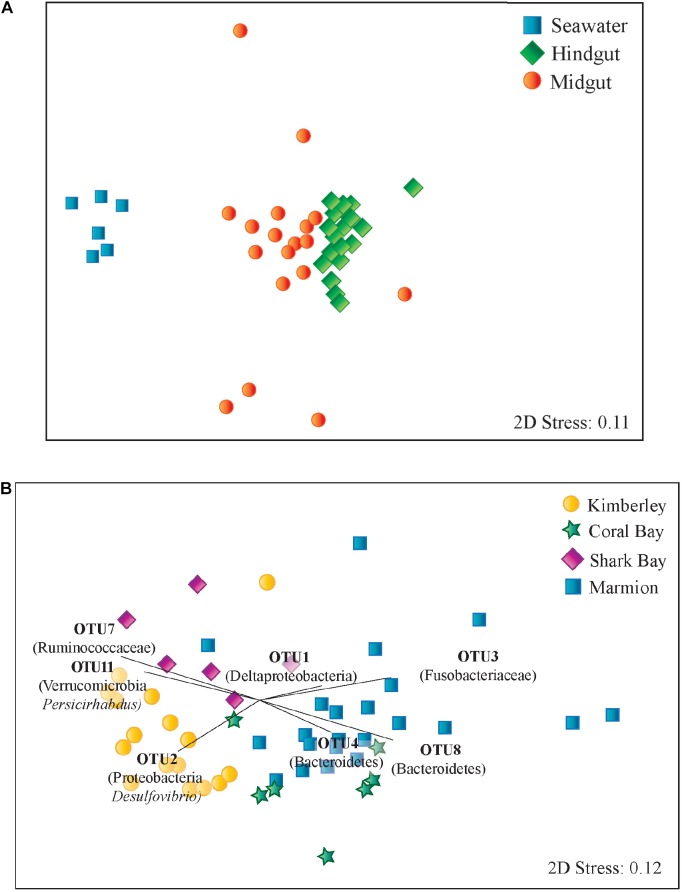
Multidimensional scaling plots based on Bray-Curtis similarity among microbial communities of *Siganus fuscescens* sampled from **(A)** different environments in Western Australia and **(B)** hindgut microbial communities. Vectors displayed indicate those OTUs identified as contributing most strongly towards hindgut microbial community similarity within sites using SIMPER analysis.

Seawater, midgut and hindgut microbial community composition were significantly different from each other (PERMANOVA, *p* < 0.01, **Supplementary Table 3**). There was also more dispersion (*p* < 0.01) in the microbial communities within midguts than the more tightly clustered hindgut and seawater communities (**Figure 3A**). Midgut samples shared an average similarity of 29.32% while hindgut samples shared an average similarity of 50.35% (SIMPER analysis). In general, OTUs from midgut regions were more closely related to sequences from environmental sources such as seawater, sediment, corals and soils, while OTUs from hindguts were more closely related to sequences from the hindguts of either herbivorous fishes or other vertebrates (**Supplementary Table 4**).

### Site Comparison of *Siganus fuscescens* Hindgut Microbial Communities

Alpha diversity was comparable within the hindguts of fish sourced from all sites for all indices, except for Shannon’s *H* diversity metric, which was significantly lower at Marmion Marine Park, located at the highest latitude, than at the Kimberley site, located at the lowest latitude (**Table 1**).

There was a significant difference in hindgut microbial community composition among sites (PERMANOVA, *p* < 0.05, Global *R* = 0.591), with no significant difference in dispersion of data within hindguts from each site (PERMDISP, *p* > 0.05). Pairwise comparisons identified differences between each pair of sites (PERMANOVA *p* < 0.05, **Figure 3B** and **Supplementary Table 3**). Bray-Curtis dissimilarity also increased with increasing distance among sites (*p* < 0.001; **Supplementary Figure 4**). Hindgut microbiomes from Marmion Marine Park and the Kimberley site had the greatest dissimilarity, as indicated by SIMPER (**Supplementary Table 5**), which was driven by differences in OTUs from a number of phyla including Bacteroidetes, Firmicutes, Fusobacteria, Proteobacteria and Verrucomicrobia.

**FIGURE 4:**
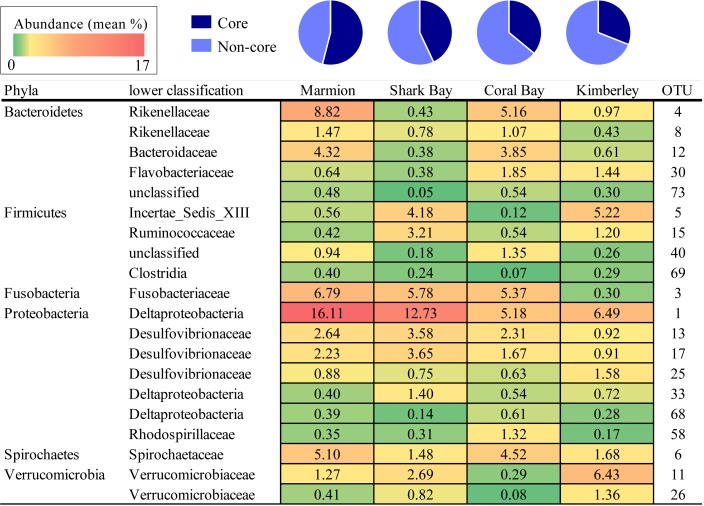
The percentage of the core OTUs (those present in all sites) from each site. Numbers are the mean percentage that each OTU contributes to the total hindgut microbiome within each site. Shading represents values from low (green) to high (red) abundance (values range between 0.05 and 16.11%).

Despite differences in community composition among sites, across all the hindgut samples from all four sites in this study, a core set of 20 OTUs was detected. These were defined as those OTUs that occurred in all samples. Most OTUs identified as ‘core’ were relatively abundant: they ranged from 0.05 to 16.11% relative abundance within each site. Overall, the core microbiome made up an average of 43% (SD 2.56) of the total community composition within hindguts from all four sites (**Figure 4**). The proportion that the core microbiome OTUs occupied within the total community at each site was largest in the high latitude site (54% in Marmion) and decreased with decreasing latitude (31% Kimberley). Deltaproteobacteria (OTU 1) was the most abundant OTU throughout, and was more abundant in the higher latitude sites than in the lower latitude sites. Marmion and Coral Bay sites shared higher proportions of Rikenellaceae (OTU 4 and 8), whereas Shark Bay and Kimberley sites had higher proportions of Incertae_Sedis_XIII (OTU 5), Ruminococcaceae (15) and Verrucomicrobiaceae (OTU 11, 26). Within the midgut (Marmion and Coral Bay populations only), there were 21 shared OTUs which made up an average of 36% of the midgut community. Within those same individuals, 101 OTUs were shared within the hindgut, and made up an average of 83% of the community (**Supplementary Figure 5**).

### Quantification of Short Chain Fatty Acids Within the Hindgut

**FIGURE 5:**
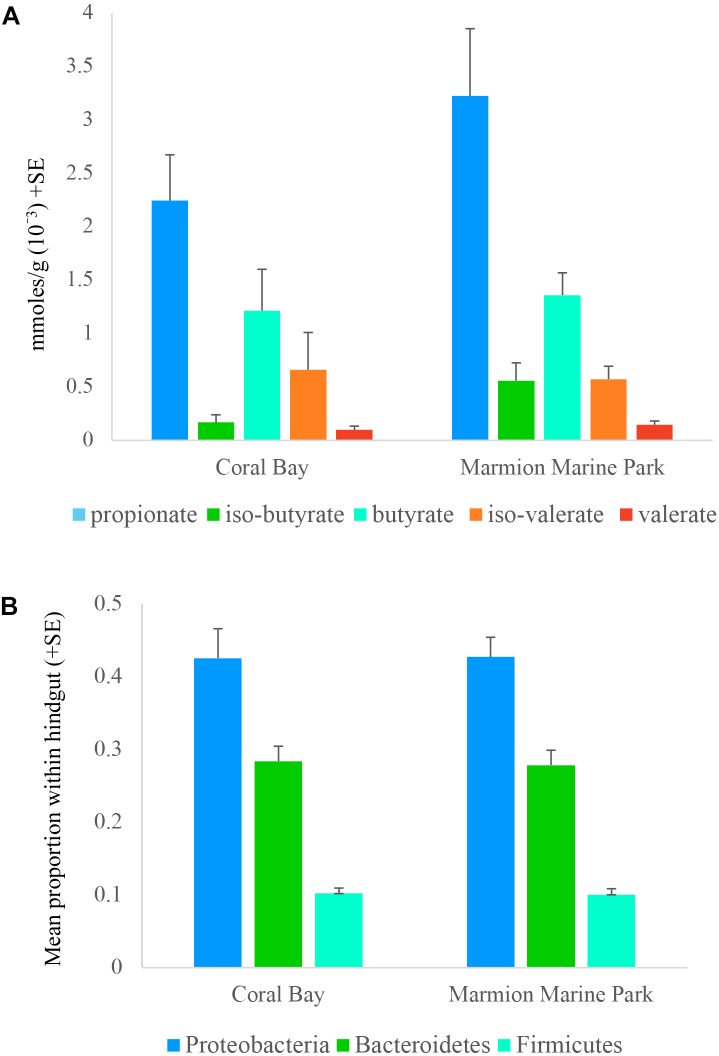
Mean **(A)** concentration of the short chain fatty acids (SCFAs) propionate, iso-butyrate, butyrate, iso-valerate, and valerate, and **(B)** proportion of bacterial phyla known to contribute to SCFA production detected within the hindgut of *Siganus fuscescens* at one site in the historical range (Coral Bay) and one site in the new temperate range (Marmion Marine Park).

Five SCFAs were quantified within the hindguts of one tropical population (Coral Bay) and one temperate population (Marmion Marine Park). Overall, for the five SCFAs quantified, each location displayed the same pattern, with the highest levels of propionate, followed by butyrate, iso-butyrate, iso-valerate and finally valerate. Acetate could not be quantified as it was detected at high concentrations in the blank and was therefore removed from the analysis. No statistical difference was identified in the quantity of each SCFA between sampling sites (two tailed *t*-tests, *p* > 0.05; **Figure 5A**) or in the total composition of SCFA (PERMANOVA, *p* > 0.05, **Supplementary Table 3**). The abundance of the three bacterial phyla known to produce SCFA (Bacteroidetes, Firmicutes, and Proteobacteria) were also similar in hindgut microbial communities from Coral Bay and Marmion Marine Park (two tailed *t*-tests, *p* > 0.05;**Figure 5B**).

## Discussion

Recent range expansion of tropical herbivorous rabbitfish (genus *Siganus*) into temperate reef ecosystems has been documented primarily in the Mediterranean Sea (*Siganus rivulatus* and *S. luridus*) (Vergés et al., 2014) and the east coast of Australia (*S. fuscescens*) (Hyndes et al., 2016). There may also be evidence of expansion beyond the recorded southern limit of this species’ range at ∼32°S on the west coast of Australia (Lenanton et al., 2017). However, confirmation of this requires demonstration that these southern populations are persistently self-recruiting, rather than observations of increases in abundance alone (e.g., Zarco-Perello et al., 2017). It is noteworthy that, while Lenanton et al. (2017) observed both reproductively mature adult and juvenile *S. fuscescens* at ∼32°S, which was in conjunction with a period of warmer than average water temperatures, such individuals were not observed when water temperatures cooled in subsequent years. Anomalously warm ocean temperatures have coincided with an increase in both the abundance of tropical herbivores and their feeding rates in these temperate areas, resulting in loss of algal cover and decreased benthic species richness of organisms such as algae, invertebrates and demersal fishes (Vergés et al., 2014; Bennett et al., 2016; Hyndes et al., 2016). On the west coast of Australia, *S. fuscescens* is found along the tropical coast of north-western Australia from the Lacepede Islands in the Kimberley region at ∼16°S through to the temperate waters of Rottnest Island and Cockburn Sound at ∼32°S (G. Moore and W. A. Museum, unpublished data; Hutchins, 1994; Travers et al., 2010). In temperate waters along this coast, observations suggest this species was not previously abundant (e.g., Ayvazian and Hyndes, 1995; Fairclough et al., 2011), but recently it has been reported more regularly (e.g., Edgar and Stuart-Smith, 2014; Lenanton et al., 2017; Zarco-Perello et al., 2017), including as far south as Geographe Bay at ∼33.5°S (Lenanton et al., 2017).

Here, we show that distinct microbial communities occur between the hindgut and midgut of *S. fuscescens* with the midgut community hosting OTUs related to environmental sources and the hindgut hosting OTUs that appear to be specialized to the role of fermentation. Also, microbial community composition of hindgut contents of *S. fuscescens* on the west coast of Australia differed significantly across the sites. Samples collected are comparable to a number of other biogeographic studies from regions that cover large distances, and often unavoidably contain temporal variation due to the challenge of sampling such distances simultaneously (e.g., Martiny et al., 2011; Luter et al., 2015; Kellogg et al., 2017). However, hindgut communities were each distinct, although samples from some sites were collected within weeks of one another. Also, all sites were dominated by a small number of abundant OTUs, despite the large geographical separation between sites and unavoidable temporal variation in sampling. Furthermore, levels of SCFAs in the hindguts of fish from the new temperate environment were similar to those from the historical tropical range. We propose that one mechanism that has enabled this rabbitfish species to successfully invade temperate reefs in Western Australia is the flexibility of their hindgut microbiota, which may be suited to the new diet and environment. The gut microbiome appears to have similar core structure and provide continued functionality, and thus shows no evidence of disturbance characterized by a loss of core microbial members, and a breakdown of community structure. This hypothesis will require further testing as new data emerge from the microbiomes of those species that are able to successfully undergo poleward range shifts.

Within this study, midgut and hindgut material were collected separately to allow for comparisons between the two gut regions. We found a more consistent microbial composition within the hindgut communities compared to those in the midgut, despite a lack of specialized structure separating the midgut and hindgut regions of *S. fuscescens*. Midguts had higher amounts of OTUs that were most similar to sequences obtained from environments that are distinct from fish GI communities such as the rectum of wild dolphins (Bik et al., 2016), seawater (Fasca et al., 2018), sediment (accession number MH312565, unpublished)^3^ and corals (Pantos et al., 2003), while hindguts had higher amounts of OTUs most similar to GI communities from either rabbitfish (Zhang et al., 2018) or other herbivorous fishes (e.g., Miyake et al., 2015). These results confirm those of Nielsen et al. (2017) in four individuals sampled from a single population of *S. fuscescens* from the east coast of Australia, and illustrate a more specialized community within the hindgut across a large portion of the coastline of Western Australia, as well as a more transient community within the midgut in comparison to the hindgut. Furthermore, we demonstrate that PCR amplification and sequencing output was more successful for hindgut than midgut samples, with success rates of approximately 90 and 57%, respectively (**Supplementary Table 2**), possibly due to higher amounts of inhibitors in undigested seaweeds. Given the higher specialization of the hindgut community to fermentation processes and the lower variability in comparison to the midgut community we argue that the hindgut region is most host-associated, and less transient, and may therefore be more suitable for comparisons across populations of grazing herbivorous fishes.

Diet is one of the leading factors that influences GI microbial communities within all vertebrates. In tropical locations across eastern Australia, adult *S. fuscescens* consume macroalgae from the genus *Sargassum* (Noda et al., 2014), as well as seagrass, including species from the genera *Cymodocea, Halodule, Halophila*, *Syringodium* and *Thalassia* (Pitt, 1997). Seagrass, as well as both red and brown algae were commonly identified within the GI tract of *S. fuscescens* in each population within this study. The specimens from this study are being included in a related large-scale analysis of herbivorous fish in Western Australia. Preliminary results indicate that *S. fuscescens* from tropical reefs primarily consume corticated foliose algae (*Dictyota* spp.) and leathery macrophytes (*Sargassum* spp.), corticated terete algae (*Hypnea* and *Laurencia*) and colonial invertebrates (hydrozoans and sponges), while dietary analysis of fish from temperate areas indicate the presence of *Hypnea* and *Laurencia*, seagrass (*Posidonia* spp.) and *Sargassum* (C. Avenant, personal communication). Bacteria commonly found on red, green and brown algae include Alphaproteobacteria, Gammaproteobacteria, Bacteroidetes and Cyanobacteria (Egan et al., 2013), and hindgut communities from the most temperate site, Marmion Marine Park, also host high amounts of several Bacteroidetes in comparison to the more tropical sites, possibly due to higher amounts of certain species of macroalgae in the diet.

Even if fish at the poleward range limit for this species are feeding on comparable diets to those in warmer waters, other factors, such as algal and seagrass chemical defenses (van Hees et al., 2017) can vary with latitude. This strongly suggests that the GI microbial community of this species is able to overcome the change in diet that this species would consume across its wide geographic distribution. Coral reef fish often exhibit a great degree of dietary complexity and plasticity (Miyake et al., 2015) and it appears that the gut microbiome of *S. fuscescens* is well suited to the diet at the poleward limit of its range, which may facilitate the success of this species beyond this limit within temperate ecosystems. Longer-term studies would allow a better understanding of any negative effects on health such as reduction in nutrient acquisition or loss of resilience to pathogens, but given that this species is fast-growing and not long-lived (Woodland, 1990), any negative effects might be expected to manifest rapidly, and therefore should be observable in the specimens examined here.

The presence of SCFAs within the hindgut organisms is an indication of microbial fermentation (Fidopiastis et al., 2006), which is a process that converts indigestible polysaccharides into an energy source that host fish can absorb (Skea et al., 2007). Fermentation rates within herbivorous fish hindguts have been shown to meet or exceed those of terrestrial mammals, illustrating that the hindgut microbial communities of fish are capable of providing an important source of energy (Mountfort et al., 2002). The quantity of SCFA present in the hindguts of herbivorous fish have been measured in several species, with the highest levels detected in *Kyphosus* spp. (family Kyphosidae) (Clements and Choat, 1997) and intermediate levels within *Hermosilla azurea* (family Kyphosidae) (Fidopiastis et al., 2006), *Naso unicornis* (family Acanthuridae) and *Siganus argenteus* (a congener of *S. fuscescens*) (Clements and Choat, 1995). Each of these herbivorous species are thought to rely on microbial fermentation to digest components of their diet (Clements and Choat, 1997; Fidopiastis et al., 2006). The quantity of propionate, butyrate and iso-butyrate within the hindgut of *S. fuscescens* in this study was comparable to those found within *H. azurea* and *S. argenteus*, whereas the levels of valerate and iso-valerate were comparable to *Kyphosus* spp., indicating that *S. fuscescens* is equally as likely to rely on microbial fermentation within the hindgut to enhance its energy supply. Indeed, this functional role of the hindgut microbial community appears to not have been reduced or otherwise impacted by the range shift of this species into temperate locations.

This study represents the first examination of the geographic variation of the gut microbiome of wild populations of the herbivorous marine rabbitfish *S. fuscescens*. In order to evaluate the capacity of this species to adapt to new environments associated with temperature-induced range shifts, the microbiome of fishes occupying cooler waters at the temperate distributional limit were compared with those occupying warmer sub-tropical and tropical waters. The south-west coast of Australia supports high levels of both floral and faunal diversity along its temperate reef systems (Wernberg et al., 2011), which are heavily influenced by the influx of warm waters from the Leeuwin Current (Feng et al., 2003). With a trend for increasing coastal water temperatures, this area has now become a hotspot for climate-mediated range shifts in marine species distributions (Wernberg et al., 2011, 2016; Hobday and Pecl, 2013; Smale and Wernberg, 2013; Cacciapaglia and van Woesik, 2015). Within this study, the overall hindgut microbial community of *S. fuscescens* appears to change significantly according to local environmental factors, which likely indicates local variations in diet, and also shows a trend toward increasing dissimilarity over a tropical to temperate latitudinal gradient, which may reflect broad scale environmental change. The presence of a core set of OTUs within the gut across all of the geographical locations sampled suggests that these members may be key contributors to both community composition and host gut function. For example, OTU 1, identified as a member of the Deltaproteobacteria genus *Desulfovibrio* was highly abundant, and members of this genus are known sulfate reducers (Zhang et al., 2018). Another abundant core OTU, OTU 6, is from the Spirochaetes family Spirochaetaceae that have recently been shown to have an active sugar-based metabolism (Stolze et al., 2018) and was previously identified as potentially being an integral component of the rabbitfish gut microbiome (Zhang et al., 2018). The structure of the hindgut microbiome also affects the quantity of SCFA produced, and similar levels of SCFA were present within the hindguts of both tropical and temperate populations, indicating no geographical effect on this important functional role of the hindgut microbial community. Overall, the hindgut microbiome of *S. fuscescens* appears to be well suited to temperate environments of south-western Australia and thus potentially also to regions beyond the southern limits of its range. This indicates that this herbivore, and possibly its congeners, may be highly successful in temperate regions if their distributions extend further poleward, and possibly alter these economically important ecosystems [e.g., the “Great Southern Reef”; (Bennett et al., 2016)].

## Data Accessibility Statement

Raw sequences have been deposited in the NCBI sequence read archive (SRA) under biosample accession numbers SAMN07425686 to SAMN07425756.

## Author Contributions

JJ, JDD, MS, MB, and MJH designed the research. JJ, JDD, MJH, DF, and MT collected the samples. JJ, JDD, MS, MCB, and MJH performed the lab work and did the analyses. All the authors contributed to interpretation of the analyses. JJ, JDD, DF, MT, and MJH wrote the paper.

## Conflict of Interest Statement

The authors declare that the research was conducted in the absence of any commercial or financial relationships that could be construed as a potential conflict of interest.

## Footnotes

1 http://www.geneious.com

2 www.ncbi.nlm.nih.gov

3 www.ncbi.nlm.nih.gov
