# Supplementary material for: The Microbiome of the Gastrointestinal Tract of a Range-Shifting Marine Herbivorous Fish
Source: Front Microbiol. 2018 Aug 28;9:2000. doi: 10.3389/fmicb.2018.02000 (PMC6121097; doi:10.3389/fmicb.2018.02000)
Supplement: Supplementary file 2 [file Data_Sheet_2.docx]

Supplementary Table 1: The mean length and weight of fish from each site. Fish from Marmion were significantly longer and heavier than all other sites (1 way ANOVA, *p* < 0.01).

| Location | Length (mm) | Weight (g) |
| --- | --- | --- |
| Marmion* | 296 ± 1.8 | 426 ± 82 |
| Kimberly | 247 ± 2.4 | 216 ± 57 |
| Coral Bay | 244 ± 3.2 | 201 ± 68 |
| Shark Bay | 240 ± 2.4 | 184 ± 56 |

Supplementary Table 2: Number of *Siganus fuscescens* specimens collected from sites in Western Australia and extracted compared with the final number of samples that successfully amplified and were analysed from each location. HG = hindgut, MG = midgut.

| Site | Collected and extracted | | Successfully sequenced | |
| --- | --- | --- | --- | --- |
|  | HG | MG | HG | MG |
| Coral Bay | 7 | 7 | 7 | 4 |
| Marmion | 24 | 11 | 22 | 10 |
| Shark Bay | 7 |  | 6 |  |
| Kimberley | 19 |  | 16 |  |
| Total | 57 | 31 | 51 | 14 |

Supplementary Table 3: Outputs of PERMDISP and PERMANOVA. Statistically significant factors (*p* <0.05) are indicated in bold, with the p value also italicised. To assess which treatments that were significantly different, pair-wise tests (PERMANOVA) were performed.

| Source  (PERMDISP) | df | F | p |
| --- | --- | --- | --- |
| Deviations from centroid | 1: 2  2: 31 | 6.860 | ***0.01*** |
| Source | Size | Average | SE |
| Midgut | 14 | 33.278 | 3.873 |
| Hindgut | 14 | 48.294 | 2.057 |
| Seawater | 6 | 33.352 | 2.158 |
| Pairwise comparisons | t | p |  |
| Hindgut, Midgut | 3.4239 | ***<0.001*** |  |
| Hindgut, Water | 1.6352 | 0.233 |  |
| Midgut, Water | 1.4242 | 0.313 |  |

| Source  (1 factor PERMANOVA) | df | Pseudo F | p |
| --- | --- | --- | --- |
| Site | 2 | 8.558 | ***0.001*** |
| Residual | 31 |  |  |
| Pairwise tests | t | p | No. Permutations |
| Hindgut, Midgut | 2.3846 | 0.001 | 998 |
| Hindgut, Water | 3.9111 | 0.002 | 989 |
| Midgut, Water | 2.7023 | 0.001 | 982 |

| Hindguts  (PERMDISP) | df | F | p |
| --- | --- | --- | --- |
| Deviations from centroid | 1: 3  2: 48 | 1.118 | 0.544 |
| Site | Size | Mean | SE |
| Marmion | 23 | 33.252 | 1.843 |
| Shark Bay | 6 | 29.392 | 2.134 |
| Coral Bay | 7 | 29.333 | 1.177 |
| Kimberley | 16 | 30.122 | 1.243 |

| Hindguts  (1 factor PERMANOVA) | df | Pseudo F | p |
| --- | --- | --- | --- |
| Site | 3 | 6.3255 | ***0.001*** |
| Residual | 48 | 1.08 | 0.32 |
| Pairwise tests | t | p | No. Permutations |
| Coral Bay, Marmion | 1.5416 | ***0.01*** | 996 |
| Coral Bay, Kimberley | 2.6034 | ***0.001*** | 994 |
| Coral Bay, Shark Bay | 2.1796 | ***0.002*** | 777 |
| Marmion, Kimberley | 3.5504 | ***0.001*** | 997 |
| Marmion, Shark Bay | 2.2153 | ***0.001*** | 996 |
| Kimberley, Shark Bay | 1.7048 | ***0.001*** | 984 |

| SCFA  (PERMDISP) | df | F | p |
| --- | --- | --- | --- |
| Deviations from centroid | 1: 1  2: 18 | 0.358 | 0.607 |
| Site | Size | Mean | SE |
| Marmion | 14 | 0.729 | 0.134 |
| Coral Bay | 6 | 0.595 | 0.126 |

| SCFA  (1 factor PERMANOVA) | df | Pseudo F | p | No. Permutations |
| --- | --- | --- | --- | --- |
| Site | 1 | 0.833 | 0.407 | 978 |
| Residual | 18 |  |  |  |

Supplementary Table 4: Nearest sequence matches in NCBI (<http://www.blast.ncbi.nlm.nih.gov>) to OTUs that are in high abundance in either the midgut (MG) or hindgut (HG) and in low abundance in the other; and those that are in highest abundance in either MG or HG and absent in the other. NCBI database accessed July 10 2018.

|  | **OTU** | **MG average abundance (%)** | **HG average abundance (%)** | **NCBI nearest match source** | **NCBI nearest match percent similarity** | **NCBI nearest match Accession No.** |
| --- | --- | --- | --- | --- | --- | --- |
| Abundant in MG and low abundance in HG | OTU 10 | 10.5 | 0.14 | Bottle nose dolphin rectum | 99% | KC258383 |
|  | OTU 36 | 4.97 | 0.03 | Freshwater fish GI tract | 96% | FR799678 |
|  | OTU 31 | 4.01 | 0.21 | Hot spring | 100% | MH388008 |
|  | OTU 104 | 2.97 | <0.01 | Pharmaceutical wastewater | 99% | MH396725 |
|  | OTU 34 | 2.42 | 0.02 | Unicorn fish GI tract | 98% | HM630195 |
|  | OTU 37 | 2.22 | 0.04 | Seawater | 100% | MH368432 |
| Top abundance in MG and absent in HG | OTU 330 | 0.9 | 0 | Coral | 99% | AF544911 |
|  | OTU 477 | 0.24 | 0 | River sediment | 95% | MH312565 |
|  | OTU 529 | 0.22 | 0 | Endolithic community | 98% | KT980550 |
|  | OTU 312 | 0.15 | 0 | Deep sea trench | 100% | LC374390 |
|  | OTU 429 | 0.23 | 0 | Seawater | 100% | LT720669 |
|  | OTU 538 | 0.1 | 0 | Soil | 100% | MH421842 |
| Abundant in HG and low abundance in MG | OTU 4 | 0.55 | 4.87 | Surgeonfish GI tract | 99% | KT952838 |
|  | OTU 6 | 0.37 | 3.52 | Angelfish GI tract | 97% | EU885092 |
|  | OTU 11 | 0.44 | 2.91 | Angelfish GI tract | 98% | EU885080 |
|  | OTU 12 | 0.48 | 2.63 | Rabbitfish GI tract | 99% | HG971010 |
|  | OTU 5 | 0.42 | 2.39 | Rabbitfish GI tract | 98% | HG971001 |
|  | OTU 14 | 0.29 | 2.27 | Cow faeces | 96% | KJ957667 |
| Top abundance in HG and absent in MG | OTU 48 | 0 | 0.48 | Cockroach GI tract | 98% | JN680635 |
|  | OTU 109 | 0 | 0.18 | Unicorn fish GI tract | 95% | HM630188 |
|  | OTU 218 | 0 | 0.08 | Horse GI tract | 95% | KY664769 |
|  | OTU 183 | 0 | 0.08 | Surgeonfish GI tract | 99% | KT952744 |
|  | OTU 106 | 0 | 0.07 | Horse GI tract | 93% | KY668176 |
|  | OTU 120 | 0 | 0.07 | Rabbitfish GI tract | 98% | HG970990 |

Supplementary Table 5: OTUs contributing to the highest amount of dissimilarity among sites, as determined by SIMPER analysis. Cumulative contribution is shown in brackets.

| OTU | Phylum | Lowest taxonomic resolution | Higher average abundance | | Percentage contribution |
| --- | --- | --- | --- | --- | --- |
|  | Marmion (M) and Kimberley (K) average dissimilarity = 59.53 | | M | K |  |
| 4 | Bacteroidetes | *Alistipes* | X |  | 1.72 |
| 14 | Firmicutes | N/A |  | X | 1.68 (3.40) |
| 3 | Fusobacteria | Fusobacteriaceae | X |  | 1.63 (5.03) |
| 2 | Proteobacteria | Desulfovibrio |  | X | 1.62 (6.65) |
| 11 | Verrucomicrobia | *Persicirhabdus* |  | X | 1.42 (8.07) |
| 1 | Proteobacteria | Deltaproteobacteria | X |  | 1.41 (9.48) |
| 5 | Firmicutes | *Arcobacter* |  | X | 1.37 (10.85) |
|  | Coral Bay (CB) - Shark Bay (SB) average dissimilarity = 56.71 | | CB | SB |  |
| 2 | Proteobacteria | Desulfovibrio | X |  | 2.48 |
| 7 | Firmicutes | Ruminococcaceae |  | X | 1.47 (3.95) |
| 5 | Firmicutes | *Arcobacter* |  | X | 1.46 (5.41) |
| 3 | Fusobacteria | Fusobacteriaceae |  |  | 1.43 (6.83) |
| 4 | Bacteroidetes | *Alistipes* | X |  | 1.33 (8.17) |
| 1 | Proteobacteria | Deltaproteobacteria |  | X | 1.29 (9.46) |
| 16 | Firmicutes | *Anaerovorax* |  | X | 1.24 (10.70) |
|  | Marmion (M) - Shark Bay (SB) average dissimilarity = 56.70 | | M | SB |  |
| 4 | Bacteroidetes | *Alistipes* | X |  | 2.00 |
| 7 | Firmicutes | Ruminococcaceae |  | X | 1.78 (3.78) |
| 3 | Fusobacteria | Fusobacteriaceae | X |  | 1.6 (5.38) |
| 16 | Firmicutes | *Anaerovorax* |  | X | 1.53 (6.91) |
| 2 | Proteobacteria | Desulfovibrio |  | X | 1.46 (8.37) |
| 5 | Firmicutes | *Arcobacter* |  | X | 1.39 (9.76) |
| 1 | Proteobacteria | Deltaproteobacteria | X |  | 1.35 (11.11) |
|  | Coral Bay (CB) – Kimberley (K) average dissimilarity = 55.91 | | CB | K |  |
| 2 | Proteobacteria | Desulfovibrio | X |  | 2.50 |
| 11 | Verrucomicrobia | *Persicirhabdus* |  | X | 1.54 (4.04) |
| 5 | Firmicutes | *Arcobacter* |  | X | 1.46 (5.50) |
| 14 | Firmicutes(100) | N/A |  | X | 1.44 (6.94) |
| 3 | Fusobacteria | Fusobacteriaceae | X |  | 1.26 (8.20) |
| 4 | Bacteroidetes | *Alistipes* | X |  | 1.23 (9.43) |
| 7 | Firmicutes | Ruminococcaceae |  | X | 1.13(10.56) |
|  | Kimberley (K) - Shark Bay (SB) average dissimilarity = 49.71 | | K | S |  |
| 14 | Firmicutes | N/A | X |  | 1.60 |
| 3 | Fusobacteria | Fusobacteriaceae |  | X | 1.54 (3.14) |
| 2 | Proteobacteria | Desulfovibrio | X |  | 1.38 (4.52) |
| 11 | Verrucomicrobia | *Persicirhabdus* | X |  | 1.23 (5.75) |
| 16 | Firmicutes | *Anaerovorax* |  | X | 1.12 (6.87) |
| 7 | Firmicutes | Ruminococcaceae |  | X | 1.06 (7.93) |
| 1 | Proteobacteria | Deltaproteobacteria |  | X | 1.04 (8.97) |
| 5 | Firmicutes | *Arcobacter* |  |  | 1.03 (10.00) |
| 17 | Proteobacteria | Desulfovibrio |  | X | 0.99 (10.99) |
|  | Coral Bay (CB) - Marmion (M) average dissimilarity = 49.65 | | CB | M |  |
| 2 | Proteobacteria | Desulfovibrio | X |  | 3.17 |
| 1 | Proteobacteria | Deltaproteobacteria |  | X | 2.01 (5.18) |
| 3 | Fusobacteria | Fusobacteriaceae |  | X | 1.67 (6.85) |
| 4 | Bacteroidetes | *Alistipes* |  | X | 1.55 (8.40) |
| 12 | Bacteroidetes | Bacteroidaceae |  |  | 1.29 (9.69) |
| 6 | Spirochaetes | *Treponema* |  |  | 1.12 (10.81) |
