# Supplementary material for: The Microbiome of the Gastrointestinal Tract of a Range-Shifting Marine Herbivorous Fish
Source: Front Microbiol. 2018 Aug 28;9:2000. doi: 10.3389/fmicb.2018.02000 (PMC6121097; doi:10.3389/fmicb.2018.02000)
Supplement: Supplementary file 3 [file Data_Sheet_3.docx]

Supplementary Figure 1: Rarefaction curves of bacteria from midguts of *Siganus fuscescens* collected from one site in the historical range (Coral Bay), shown in green, and one site in the new temperate range (Marmion Marine Park), shown in blue.

Supplementary Figure 2: Rarefaction curves of bacteria from hindguts of *Siganus fuscescens* collected from the Kimberley (yellow), Coral Bay (green), Shark Bay (red) and Marmion Marine Park (blue).

Supplementary Figure 3: Rarefaction curves of bacteria from seawater collected near *Siganus fuscescens* populations from one site in the historical range (Coral Bay), shown in green, and one site in the new temperate range (Marmion Marine Park), shown in blue.

Supplementary Figure 4: Relationship between *Siganus fuscescens* hindgut microbial community similarity (Bray-Curtis) and distance (km) between samples. Microbial similarity significantly decreased with increasing distance (*p*<0.001).


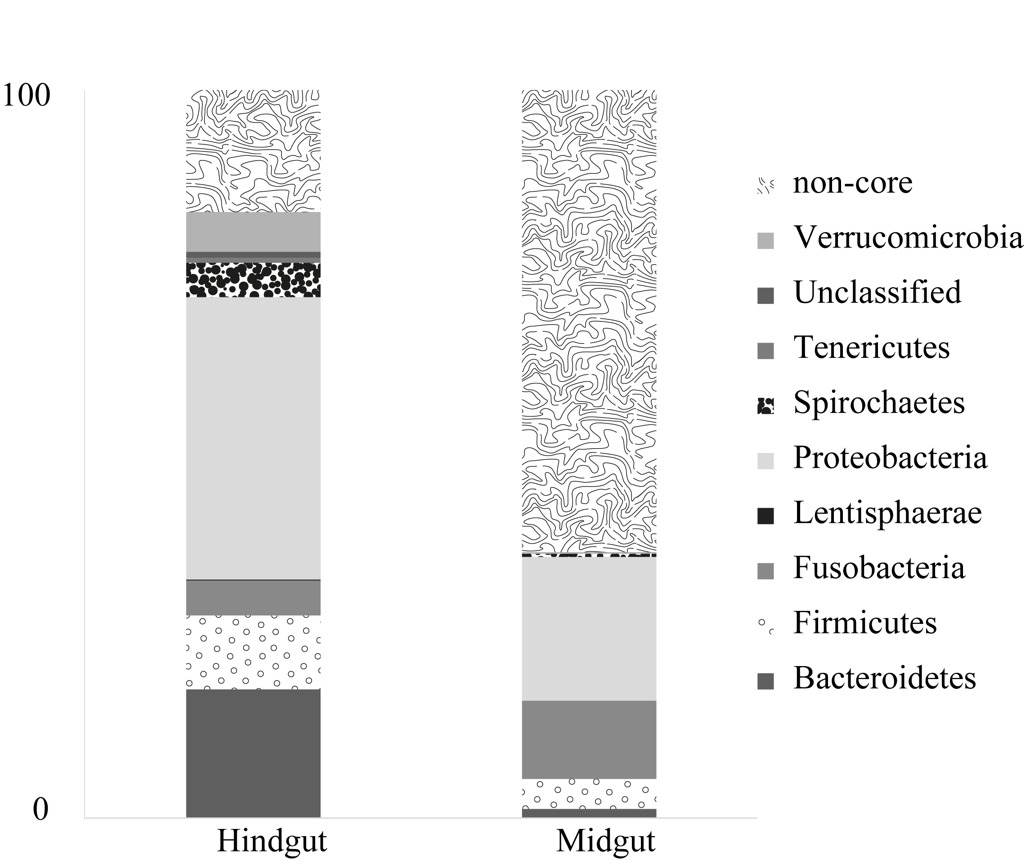


Supplementary Figure 5: Mean percent of each Phyla within the core microbiome of the hindgut and midguts of *Siganus fuscescens* from Marmion Marine Park and Coral Bay.
